# Disease Severity Determines Timing of Initiating Continuous Renal Replacement Therapies: A Systematic Review and Meta-Analysis

**DOI:** 10.3389/fmed.2021.580144

**Published:** 2021-11-18

**Authors:** Zi-Jing Xia, Lin-ye He, Shu-Yue Pan, Rui-Juan Cheng, Qiu-Ping Zhang, Yi Liu

**Affiliations:** ^1^Department of Rheumatology and Immunology, West China Hospital, Sichuan University, Chengdu, China; ^2^Department of Thyroid and Parathyroid Surgery, West China Hospital, Sichuan University, Chengdu, China; ^3^Department of Rheumatology and Immunology, Chengdu Fifth People's Hospital, Chengdu, China

**Keywords:** continuous renal replacement therapies, CRRT, timing, acute kidney injury, AKI

## Abstract

**Background:** Timing of initiating continuous renal replacement therapies (CRRTs) among the patients with acute kidney injury (AKI) in intensive care units (ICU) has been discussed over decades, but the definition of early and late CRRT initiation is still unclear.

**Methods:** The English language randomized controlled trials (RCTs) and cohort studies were searched through MEDLINE, EMBASE, and Cochrane Library on July 19, 2019, by the two researchers independently. The study characteristics; early and late definitions; outcomes, such as all-cause, in-hospital, 28- or 30-, 60-, 90-day mortality; and renal recovery were extracted from the 18 eligible studies. Pooled relative risk ratios (RRs) and 95% CIs were estimated with the fixed effects model and random effects model as appropriate. This study is registered with PROSPERO (CRD 42020158653).

**Results:** Eighteen studies including 3,914 patients showed benefit in earlier CRRT (*n* = 1,882) over later CRRT (*n* = 2,032) in all-cause mortality (RR 0.78, 95% CI 0.66–0.92), in-hospital mortality (RR 0.81, 95% CI 0.67–0.99), and 28- or 30-day mortality (RR 0.81, 95% CI 0.74–0.88), but in 60- and 90-day mortalities, no significant benefit was observed. The subgroup analysis showed significant benefit in the disease-severity-based subgroups on early CRRT initiation in terms of in-hospital mortality and 28- or 30-day mortality rather than the time-based subgroups. Moreover, early CRRT was found to have beneficial effects on renal recovery after CRRT (RR 1.21, 95% CI 1.01–1.45).

**Conclusions:** Overall, compared with late CRRT, early CRRT is beneficial for short-term survival and renal recovery, especially when the timing was defined based on the disease severity. CRRT initiation on Acute Kidney Injury Network (AKIN) stage 1 or Risk, Injury, Failure, Loss of kidney function, and End-stage kidney disease (RIFLE)-Risk or less may lead to a better prognosis.

## Introduction

Acute kidney injury (AKI) is one of the most common complications in the intensive care unit (ICU) with a 10–30% rate of hospitalization (1–3). Along with a 2–5 times risk of mortality (4)of non-AKI patients, AKI has been considered the most dangerous killer in the ICU.

Renal replacement therapy (RRT), along with other general and medical therapy, is now the primary management strategy of patients with AKI. With basic modes, such as continuous renal replacement therapy (CRRT) and intermittent renal hemodialysis (IHD), this strategy can replace and support renal function during the AKI course. With appropriate initiation timing, CRRT can theoretically be more suitable for maintaining the hemodynamic stability of patients and removing certain small molecular toxins, which can significantly improve the prognosis and reduce the mortality of the patients with AKI (5–7).

At present, despite the improvement in CRRT technology, the timing of CRRT initiation is still under intense discussion and the evidence guiding clinicians in initiating CRRT in critical diseases is still limited. One of the most important barriers is that the studies on early and late CRRT are not consistent; that is, different early and late definitions that reflect the diversity of time factors, biochemical indicators, and clinical parameters, were all used to balance the risk of initiating CRRT and the benefits of supporting renal function during critical illness. This can be a major cause of discrepancy in conclusions drawn by different studies, for example, “early” in one research can be recognized as “late” in another.

Earlier initiation of CRRT might provide better control of acid–base and electrolyte balance. Moreover, it can be more helpful in maintaining hemodynamic stability, reducing risks of other potential complications of AKI (8). While early initiation of CRRT can also increase the unnecessary financial burden of patients with AKI, it can increase the risk of coagulation-anticoagulant disorder and even delay the recovery of renal function, which may negatively affect the prognosis of patients (9). On the contrary, late initiation of CRRT may provide more time to the patients with AKI for hemodynamic optimization before CRRT or even avoid the need for CRRT and its associated complications (10). Some systematic reviews and meta-analyses have addressed these issues and provided a hint that the patients with AKI accepting early CRRT appear to benefit from it (11, 12). However, these studies still used a mixture of early and late when defining the timing of CRRT initiation. No clear rule was used in these studies to measure the timing of CRRT initiation.

This systematic review was conducted to assess the effectiveness of the different initiation timing of CRRT among the patients with AKI in the ICU and try to address the use of inconsistent definitions of early and late in different studies by subgroup analysis.

## Methods

### Overall

This systematic review with individual patient data meta-analysis was registered on PROSPERO (CRD 42020158653) and followed a prespecified analysis plan (https://www.crd.york.ac.uk/PROSPERO/). This study is reported in accordance with the Preferred Reporting Items for a Review and Meta-analysis of Individual Participant Data. The PICO principle of this study can be summarized as: Patient: patients with AKI requiring hemodialysis in ICU; Intervention: early CRRT initiation; Comparison: delayed CRRT initiation; Outcome: morality decrease.

### Search Strategy

In accordance with the Preferred Reporting Items for Systematic Reviews and Meta-Analyses (PRISMA) guidelines, we conducted this systematic review and meta-analysis to find out the effect of early and late initiation of CRRT on the outcomes of the patients with AKI requiring dialysis (13). PubMed, MEDLINE, and EMBASE databases were searched for articles comparing early and late initiation of CRRT published up to July 2019, using the search expression of (Acute kidney failure OR Acute kidney tubule necrosis OR acute kidney OR acute renal) AND (Continuous Renal replacement therapy OR dialysis OR dialyzed OR dialyzing OR hemodialysis OR hemofiltration) AND (Time to treatment OR Time OR Early intervention OR Early OR Earlier OR Timing OR Accelerated OR Accelerating OR Acceleration OR Late) AND (Critical Care OR Intensive Care Unit OR ICU).

### Inclusion Criteria

The articles were included if they meet the following inclusion criteria:

(1) Compare “early” and “late” initiation of CRRT directly on the patients with AKI aged >14 years.
(2) Provided a clear definition of “early” and “late” initiation and outcome measurements.
(3) Provided effective data on necessary basic characteristics and outcomes.

### Exclusion Criteria

The articles were excluded if they met any of these exclusion criteria:

(1) Studies did not focus on the patients with AKI requiring hemodialysis.
(2) Studies that included patients aged <14 years.
(3) Studies that included patients with chronic kidney disease or end-stage renal disease.
(4) Studies with unoriginal data.

Only randomized and cohort studies were included. Both abstracts and full papers were used for data syntheses and quality assessment. The authors of abstracts were contacted for details if possible. Two researchers independently conducted the study searching and screening and cross-checked them after completion. If there were any differences, they would be settled through discussion or by a third investigator.

### Data Extraction

For each article, data of basic characteristics, such as leading author, publication year, study design, study period, total number of patients, mean age, sex, and biochemical laboratory tests at the initiation of CRRT; main characteristics, such as type of patient setting, definition of early and late initiation of CRRT; and main study outcomes were extracted separately by the two reviewers.

The main outcomes were as follows:

(1) All-cause mortality (without time limitation).
(2) In-hospital, 28- or 30-, 60-day, and 90-day mortality.
(3) Rate of renal recovery after treatment.

### Quality Assessment

The quality of randomized controlled trials (RCTs) was evaluated with Cochrane review tools recommended by the Cochrane Handbook (14) and the Newcastle–Ottawa Scale for the observational studies (15).

### Subgroup Analysis

The subgroup analyses of the varying definitions of early and late initiations of CRRT from each study were conducted for the analyses of in-hospital mortality and 28- or 30-day mortality.

1. Studies were divided into the following subgroups based on the natural definition of early and late initiation:
  (1). Time-based studies: these studies defined early and late initiations as the time period from ICU admission, undertaking surgical or diagnosis with AKI (defined as T_0_), to the initiation of CRRT.
  (2). Disease severity-based studies: these studies defined early and late initiations based on the disease severity factors [such as biochemical indicator levels, urine output before dialysis, Acute Kidney Injury Network (AKIN) stages, Risk, Injury, Failure, Loss of kidney function, and End-stage kidney disease (RIFLE) classification, or Sequential Organ Failure Assessment scores].
2. The subgroups were re-grouped based on an introduced rule:
  (1) Based on AKIN stage of the patient or RIFLE classification: using AKIN stages and RIFLE classification provided in the included studies, the patients were re-grouped into early (defined as AKIN 1 stage or RIFLE-Risk or less) and late (defined as AKIN stages 2–3 or RIFLE-Injury or Failure) groups. The studies that did not directly provide AKIN stages or RIFLE classification but can be judged based on their definition of early and late initiation were also included.
  (2) Based on the enrollment time period of the patient from T_0_ to initiation of CRRT: the objects were re-grouped into early (defined as CRRT initiation ≤ 48 h from T_0_) and late (defined as CRRT initiation >48 h from T_0_) groups.

### Statistical Analyses

We assessed heterogeneity using *I*^2^ and τ^2^ statistics [*I*^2^ values ranged from 0 to 100%, with 0% as no observed heterogeneity, 0–74% acceptable heterogeneity, ≥75% as high heterogeneity (14)]. No meta-regression was conducted because of the small number of studies enrolled. A random effects model was used in the meta-analysis of studies with high heterogeneity (*I*^2^ ≥ 50%), and a fixed effects model was chosen when the heterogeneity was low (*I*^2^ < 50%). A Mantel–Haenszel method was used to calculate the overall risk ratios (RRs) and 95% CIs). A value of *p* ≤ 0.05 was considered statistically significant. Moreover, we conducted sensitivity analyses to assess the influence of clinical factors and measures of study quality on heterogeneity in the selected studies. By removing one study at a time from the analysis, the influence of each study on the conclusion was estimated, and the degree to which the pooled effect size changed was determined. A study was considered influential if its exclusion had an effect estimate of at least 20% or changed the conclusion. Publication bias and other reporting biases were then assessed with a funnel plot, by plotting the SE against the log RR, using the Egger test (16). No significant publication bias occurred when the shape of the funnel plot was symmetrical. All our analyses were performed with Review Manager version 5.2 (RevMan; Copenhagen: The Nordic Cochrane Center, the Cochrane Collaboration, 2014, London, UK) software.

## Results

### Study Characteristics

**Figure 1:**
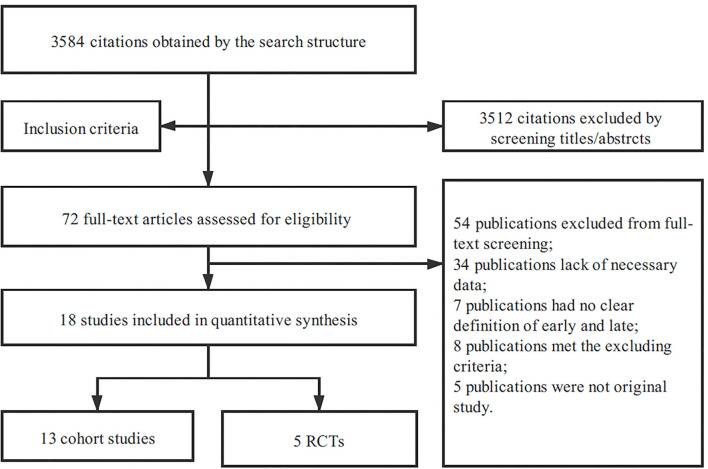
Flowchart of study selection.

As shown in Figure 1, we screened 2,019 unique articles, of which 67 were considered for full-text reviews. A total of 18 articles (14–31) that included five RCTs (17–21), five prospective cohort studies (22–26), and eight retrospective cohort studies (27–34), were eligible for the final meta-analysis (Table 1). The studies were conducted from 1989 to 2017 and analyzed a total of 3,914 patients with AKI requiring CRRT. Table 2 shows the definition of early and late CRRT initiation and the event counts of major endpoints in each study. The study conducted by Bouman et al. (17) included three cohorts, namely, early high-volume hemofiltration, early low-volume hemofiltration, and late low-volume hemofiltration, and to ensure comparability, the early high-volume hemofiltration cohort was excluded.

**Table 1:** Summary of the characteristics of studies included.

**References**	**Country**	**Study design**	**Study period**	**Cause of AKI**	***N***	**Male (%)**	**Mean age**	**Study quality**
Bouman et al. (17)	Netherlands	RCT	1998–2000	All cause	71	42 (59.2)	69	High
Sugahara and Suzuki (18)	America	RCT	1995–1997	Cardiac surgery (CABG)	28	18 (64.3)	64	Low
Jun et al. (19)	Australia	RCT	NA	All cause	439	282 (64.2)	64	High
Combes et al. (20)	America	RCT	2009–2012	Cardiac surgery	224	177 (79.0)	59	High
Zarkbock et al. (21)	Germany	RCT	2013–2015	All cause	231	146 (63.2)	67	High
Demirkiliç et al. (22)	Turkey	PC	1992–2001	Cardiac surgery	61	48 (78.7)	NA	9
Oh et al. (23)	Korea	PC	2009–2011	Septic	210	126 (60.0)	62	9
Campos et al. (24)	Germany	PC	2006–2011	Cardiac surgery	30	NA	65	7
Oh et al. (25)	Korea	PC	2008–2013	Septic	60	52 (86.7)	66	8
Park et al. (26)	Korea	PC	2009–2013	All cause	607	365 (60.1)	74	7
Gettings et al. (27)	America	RC	1989–1997	Trauma	100	79 (79.0)	45	9
Elahi et al. (28)	United kingdom	RC	2002–2003	Cardiac surgery	64	48 (75.0)	70	9
Iyem et al. (29)	Turkey	RC	2004–2007	Cardiac surgery (CABG)	185	68 (36.8)	63	9
Shiao et al. (30)	Taiwan	RC	2002–2005	Surgery	98	57 (58.2)	66	8
Shum et al. (31)	China	RC	2008–2011	Septic	120	77 (64.2)	73	8
Wu et al. (32)	China	RC	2008–2010	Surgery	73	48 (65.8)	61	8
Tian et al. (33)	China	RC	2009–2011	Septic	100	69 (69.0)	51	8
Christiansen et al. (34)	Denmark	RC	2005–2015	All cause	1,213	838 (69.1)	68	7

*AKI, acute kidney injure; CABG, coronary artery bypass graft; NA, not available; RCT, randomized controlled trial*.

**Table 2:** Definition of early and late continuous renal replacement therapy (CRRT) initiation and event counts of major endpoints in each study.

**References**	**Definitions of early and late**		**Endpoint**	**Counts**
	**Early**	**Late**		
Bouman et al. (17)	within 12 h if urine output <30 ml/h	Urea >40 mmol/L or *K* > 6.5 mmol/L	In-Hospital mortality	I:18/35 C:14/36
			28-or 30-day mortality	I:11/35 C:9/36
			60-day mortality	I:16/35 C:13/36
			90-day mortality	I:16/35 C:13/36
Sugahara and Suzuki (18)	Urine output <30 mL/h	Urine output <20 ml/h	14-day mortality	I:2/14 C:14/2
Jun et al. (19)	Within 46 h from randomization	Over 46 h from randomization	28- or 30-day mortality	I:82/219 C:84/220
			90-day mortality	I:91/219 C:102/220
Combes et al. (20)	SAPS II ≤ 45	SAPS II > 45	In-hospital mortality	I:50/112 C:44/112
			28- or 30-day mortality	I:40/112 C:40/112
			60-day mortality	I:48/112 C:42/112
			90-day mortality	I:51/112 C:43/112
			Renal recovery after CRRT	I:60/112 C:66/112
Zarkbock et al. (21)	Within 8 h from diagnosis with AKIN2 stage AKI	Within 12 h from diagnosis with AKIN stage 3 AKI	28- or 30-day mortality	I:34/112 C:48/119
			60-day mortality	I:43/112 C:60/119
			90-day mortality	I:44/112 C:65/119
			Renal recovery after CRRT	I:60/112 C:46/119
Demirkiliçet al. (22)	SCr > 400 μmol/L, *K* > 5.5 mmol/L	Oliguria	In-hospital mortality	I:6/27 C:29/34
Oh et al. (23)	Within 48 h from start time of vasopressor infusion	Over 48 h from start time of vasopressor infusion	28- or 30-day mortality	I:67/105 C:92/105
Cardenas et al. (24)	Within 48 h from cardiac surgery	Over 48 h from cardiac surgery	In-hospital mortality	I:9/15 C:10/15
Oh et al. (25)	Within 26.4 h from the start of EGDT and CRRT	Over 26.4h from the start of EGDT and CRRT	28- or 30-day mortality	I:9/30 C:17/30
Park et al. (26)	Median 6 h urine output ≥ 0.24 mL/kg/h	Median 6 h urine output <0.24 mL/kg/h	In-hospital mortality	I:121/303 C:182/304
			28- or 30-day mortality	I:163/303 C:204/304
			60-day mortality	I:170/303 C:218/304
			90-day mortality	I:176/303 C:224/304
Gettings et al. (27)	Urea <21.4 mmol/L	Urea >21.4 mmol/L	In-hospital mortality	I:25/41 C:47/59
			Renal recovery after CRRT	I:16/16 C:11/12
Elahi et al. (28)	Urine output <100 ml in 8 h	SCr > 250 mmol/L, *K* > 6 mmol/L	In-hospital mortality	I:8/28 C:12/36
Iyem et al. (29)	RRT on admission	After 48 h when anuric	In-hospital mortality	I:5/95 C:6/90
Shiao et al. (30)	RIFLE criteria (risk)	RIFLE criteria (injury and failure)	In-hospital mortality	I:22/51 C:35/47
			28- or 30-day mortality	I:14/51 C:19/47
			60-day mortality	I:23/51 C:32/47
			90-day mortality	I:29/51 C:38/47
			Renal recovery after CRRT	I:22/51 C:10/47
Shum et al. (31)	RIFLE criteria (risk)	RIFLE criteria (injury and failure)	In-Hospital mortality	I:17/31 C:48/89
			28- or 30-day mortality	I:15/31 C:43/89
			90-day mortality	I:19/31 C:49/89
			Renal recovery after CRRT	I:15/16 C:44/46
Wu et al. (32)	RIFLE criteria (risk)	RIFLE criteria (injury and failure)	28- or 30-day mortality	I:8/20 C:41/53
			90-day mortality	I:10/20 C:45/53
Tian et al. (33)	AKIN stage 1	AKIN stage ≥2	28- or 30-day mortality	I:6/23 C:43/77
			Renal recovery after CRRT	I:18/23 C:26/77
Christiansen et al. (34)	AKIN stage 2 or less	AKIN stage 3	90-day mortality	I:326/621 C:269/592

*I, intervention group; C, control group; AKIN criteria (stage 1), serum creatinine by 1.5–1.9 times the baseline or ≥0.3 mg/dl (≥26.5 μmol/L) increase or urine output <0.5 mL/kg/h × 6–12 h; stage 2, increase in serum creatinine by 2.0–2.9 times the baseline or urine output <0.5 mL/kg/h over 12 h; stage 3, serum creatinine by 3 times the baseline or ≥4.6 mg/dl (≥353.6 μmol/L) increase or initiation of RRT or in patients aged <18 years a decrease in eGFR <35 ml/min/1.73 m^2^. RIFLE criteria (Risk), increase in serum creatinine by 1.5 times or urine output <0.5 ml/kg/h × 6 h; injure, increase in serum creatinine by 2 times or urine output <0.5 ml/kg/h × 12 h; failure, increase in serum creatinine by 3 times or urine output <0.3 ml/kg/h × 24 h. AKIN, Acute Kidney Injury Network; CRRT, continuous renal replacement therapy; RIFLE, Risk, Injury, Failure, Loss of kidney function, and End-stage kidney disease; EGDT, early goal-directed therapy*.

**Figure 2:**
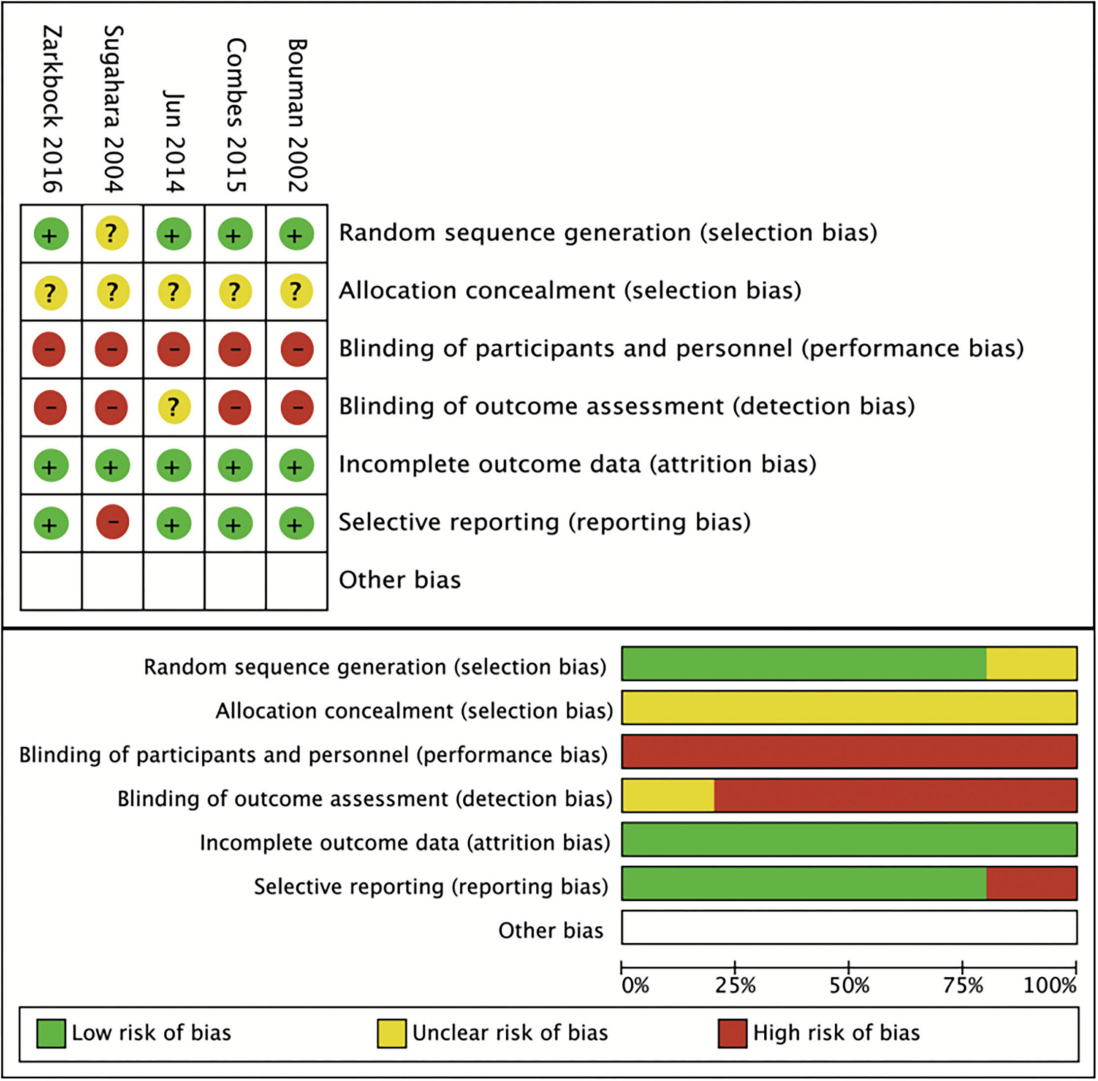
Risk of bias assessment of the included randomized controlled trials (RCTs).

**Figure 3:**
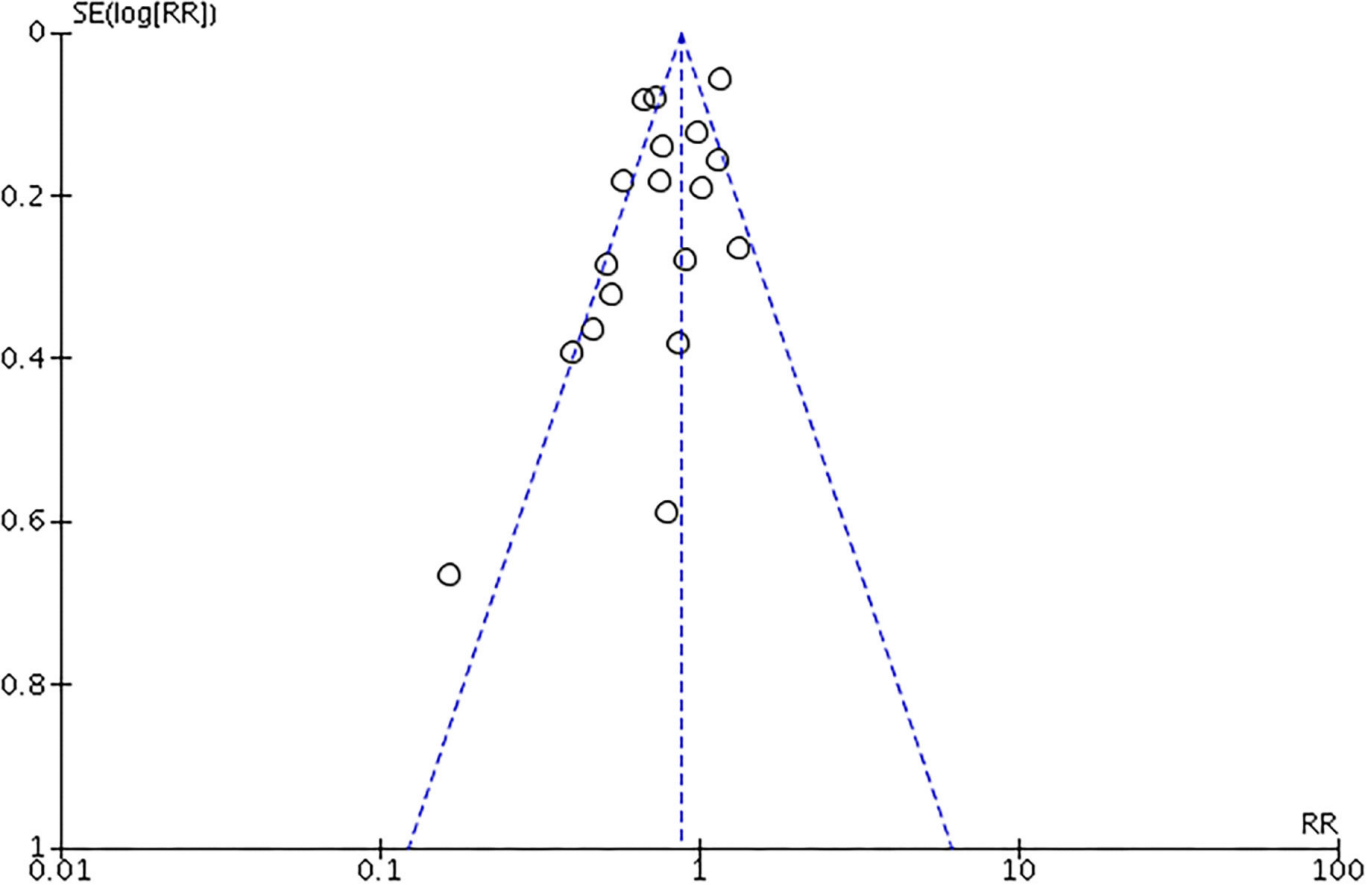
Assessment of the publication bias with funnel plots. *X*-axis for risk ratio (RR) of all-cause mortality. *Y*-axis for 8 SE of log RR.

The included RCTs were divided into low and high quality using a cutoff with three of six domains of bias in the quality assessment tool, and only the study conducted by Sugahara and Suzuki (18) (Figure 2) had low quality. All non-RCTs included were considered to have high methodological quality with a recommended cutoff score of 5 (35) (Table 1). Figure 3 shows the evaluated conclusion of publication bias. It can be speculated that no significant publication bias exists because the shape of the funnel plot was symmetrical. However, the publication bias was still of concern because only a small number of studies met our inclusion criteria.

### Effects of Early vs. Late CRRT Initiation on Mortality

**Figure 4:**
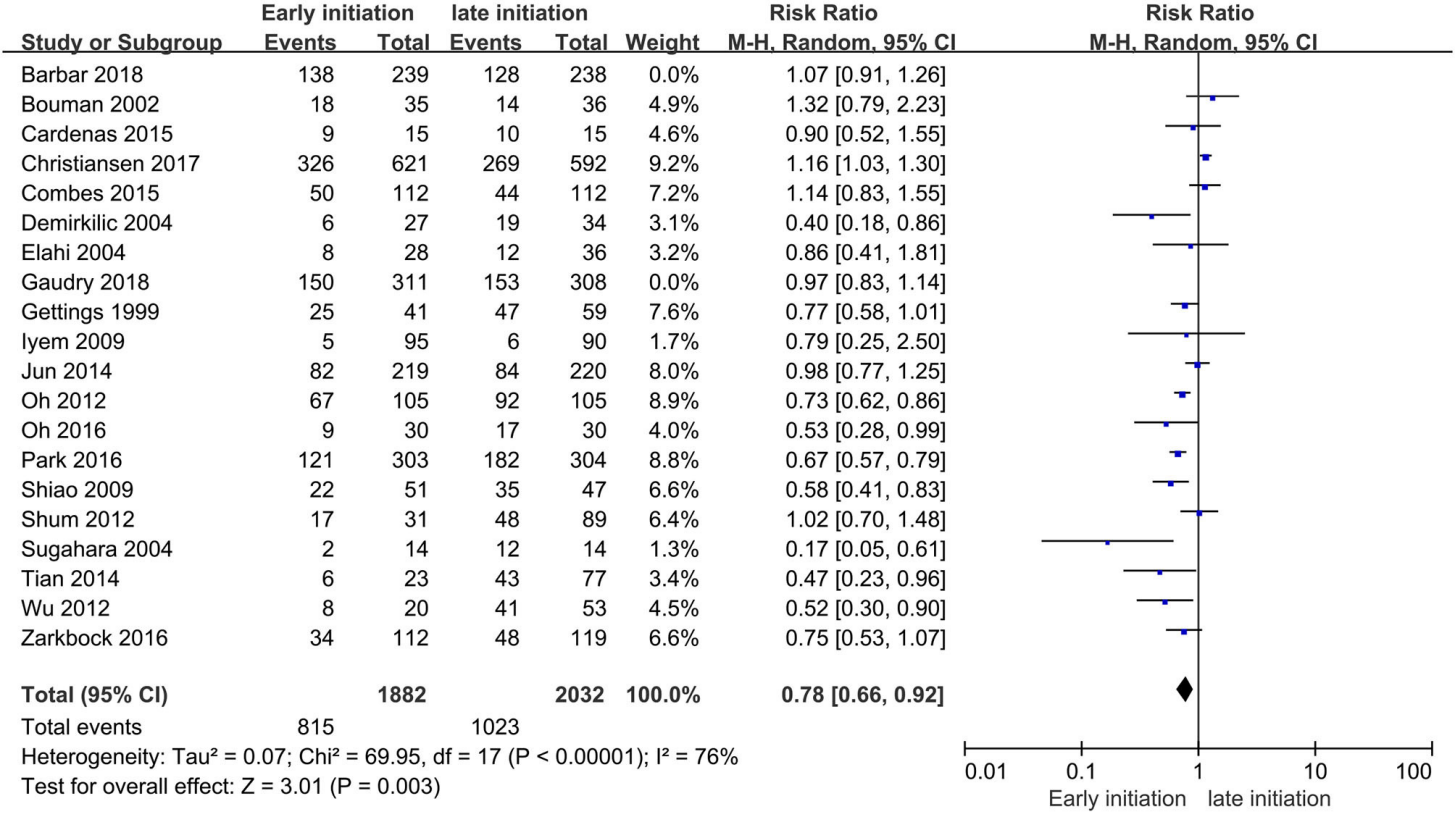
Forest plot for all-cause mortality of all studies.

The overall pooled mortalities of enrolled studies were 43.3% (815 of 1,882) and 50.3% (1,023 of 2,032) in the early and late groups, respectively. A significant benefit was found in the early group (RR 0.78, 95% CI 0.66–0.92, *p* = 0.003, Figure 4).

Of the 18 studies, 10 reported data on in-hospital mortality, that is, 38.1% (281 of 738) in the early group and 57.3% (471 of 822) in the late group, and the benefit of early initiation was significant (RR 0.81, 95% CI 0.67–0.99, *p* = 0.04). In addition, 11 of 18 studies provided data on 28- or 30-day mortality, with 43.1% (449 of 1,041) in the early group and 53.7% (640 of 1,504) in the late group reaching this event. Similar benefit was found in the early group (RR 0.81, 95% CI 0.74–0.88, *p* < 0.001).

**Figure 5:**
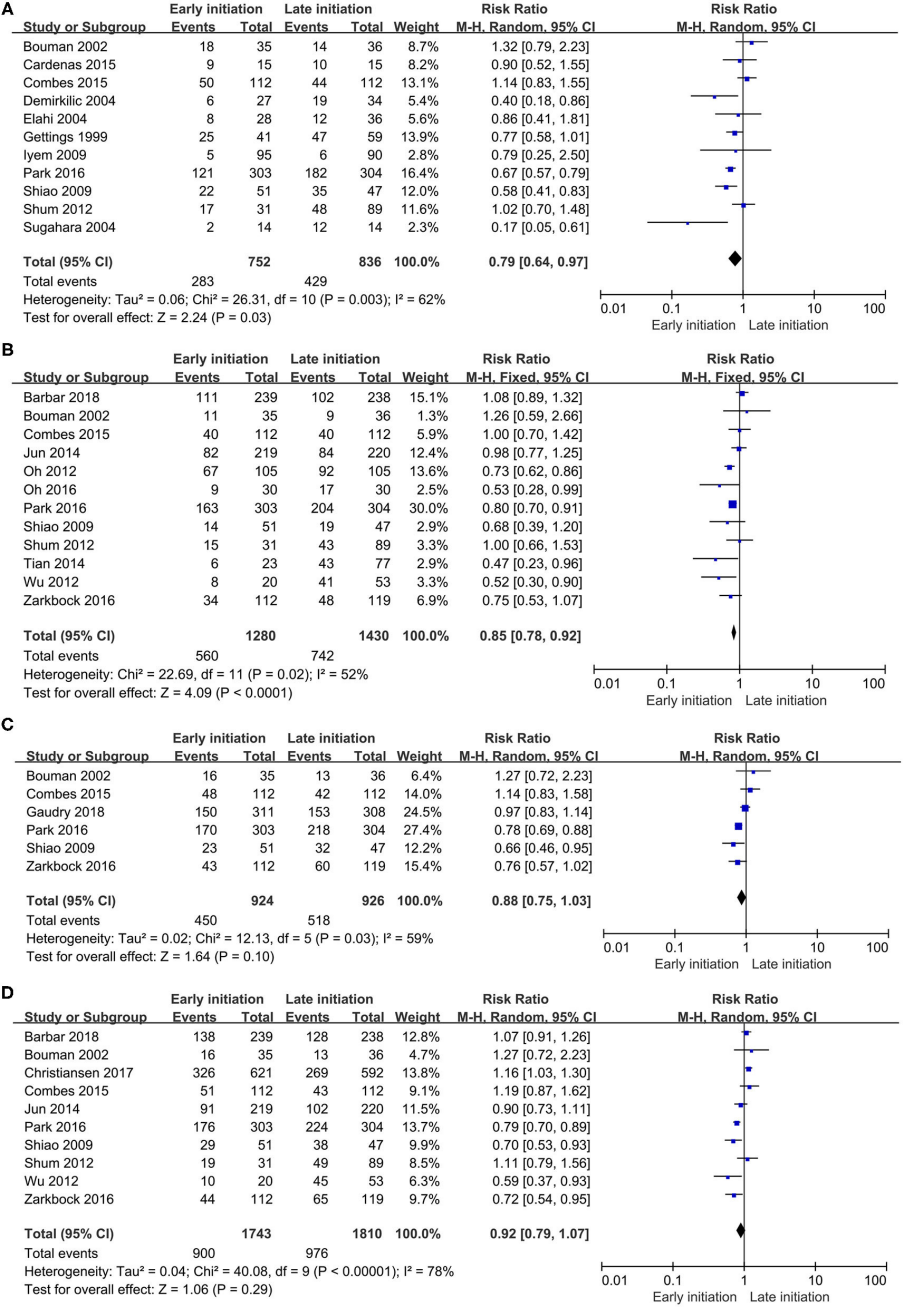
Forest plot. **(A)** In-hospital mortality. **(B)** 28- or 30-day mortality. **(C)** 60-day mortality. **(D)** 90-day mortality.

However, no specific positive effect was found in 60- and 90-day mortality. We believed that substantial heterogeneity existed among these studies in most of the analyses, except for analysis on 28- or 30-day mortality (*I*^2^ = 32%, *p* = 0.1, Figure 5).

### Effects of Early vs. Late CRRT Initiation on Renal Recovery

**Figure 6:**
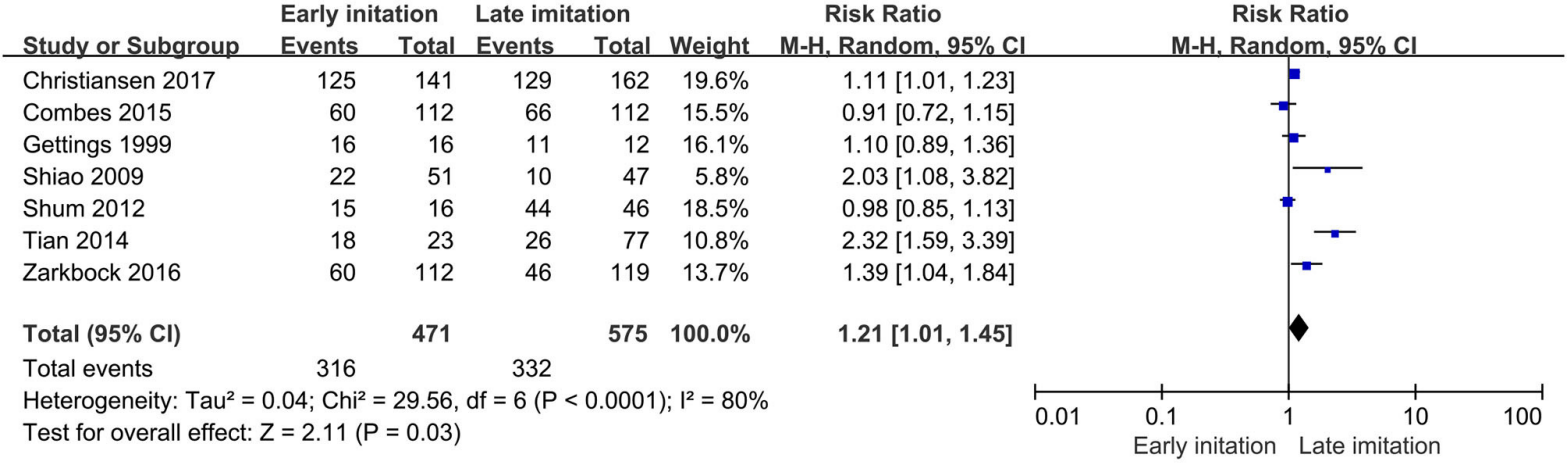
Forest plot for renal recovery after continuous renal replacement therapy (CRRT).

Of the 18 studies, seven reported data on renal recovery after CRRT. For this event, 316 of 471 patients (67.1%) in the early group and 332 of 575 patients (57.7%) in the late group reached a renal recovery after CRRT. The pooled analysis demonstrated a significant difference in this outcome between the two groups (RR 1.21, 95% CI 1.01–1.45, *p* = 0.03), while the heterogeneity was still significant (*I*^2^ = 80%, *p* < 0.001, Figure 6).

### Subgroup Analysis

To investigate factors of heterogeneity, the subgroup analyses based on the early and late definitions were conducted.

The researchers of five studies used the time-based separations to define the early and late cohorts (19, 23–25, 29), others used disease severity-related standards for their definition (18, 20, 22, 26–28, 30–34), while Bouman et al. (17) and Zarkbock et al. (21) used a time-and-disease-severity combined definition to separate the early and late cohorts.

**Figure 7:**
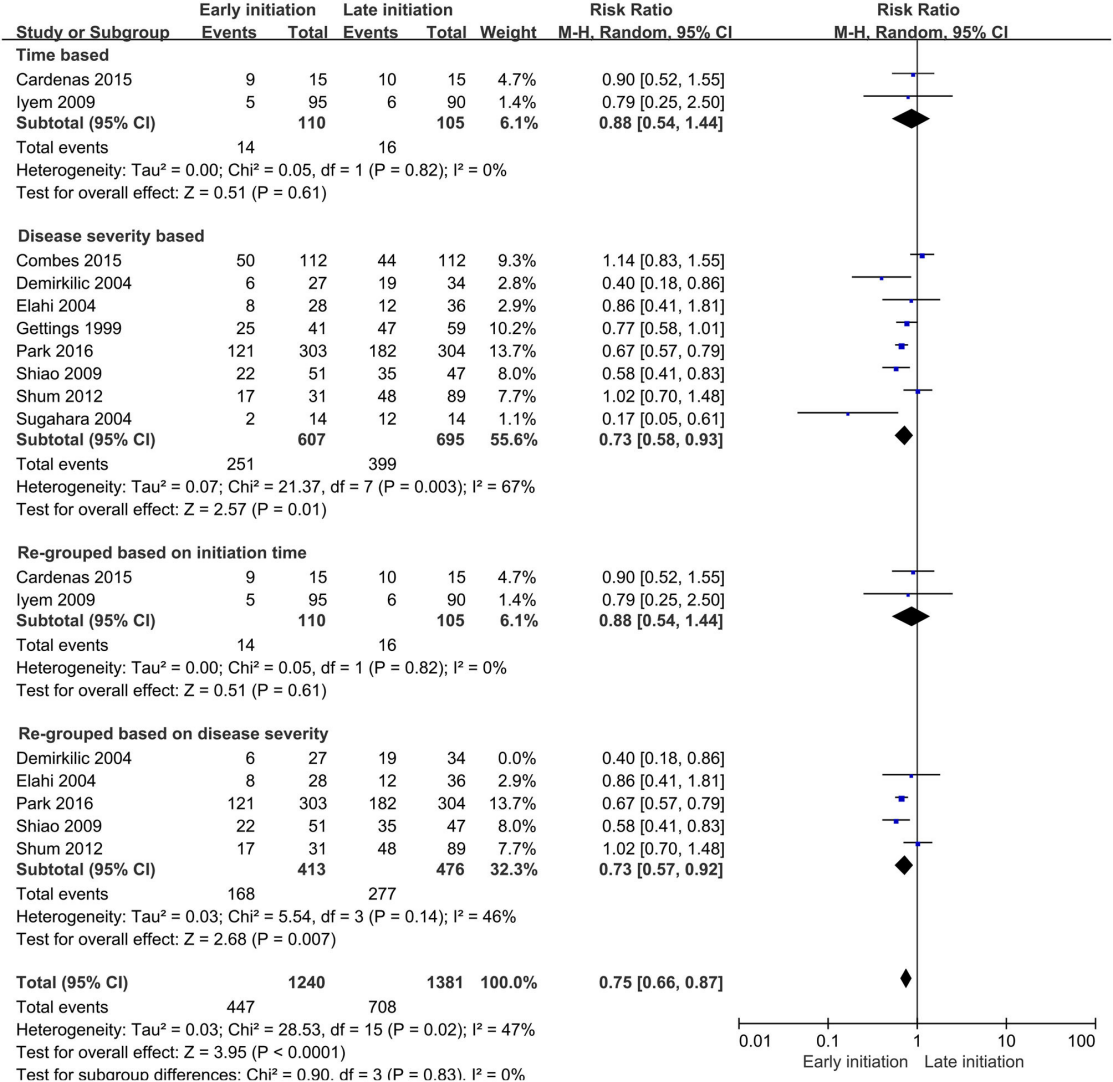
Forest plot for the subgroup analysis on in-hospital mortality.

The significant differences in the effects of timings of CRRT initiation on in-hospital mortality were found in the disease severity-based study subgroups (RR 0.73, 95% CI 0.58–0.93, *p* = 0.01) and the disease severity re-grouped subgroups (RR 0.69, 95% CI 0.55–0.88, *p* = 0.003), but no difference was found either in the time-based subgroup or time-based re-grouped subgroup. The subgroup difference analysis indicated that the heterogeneity might not exist between the subgroups (*I*^2^ = 0%, *p* = 0.8, Figure 7).

**Figure 8:**
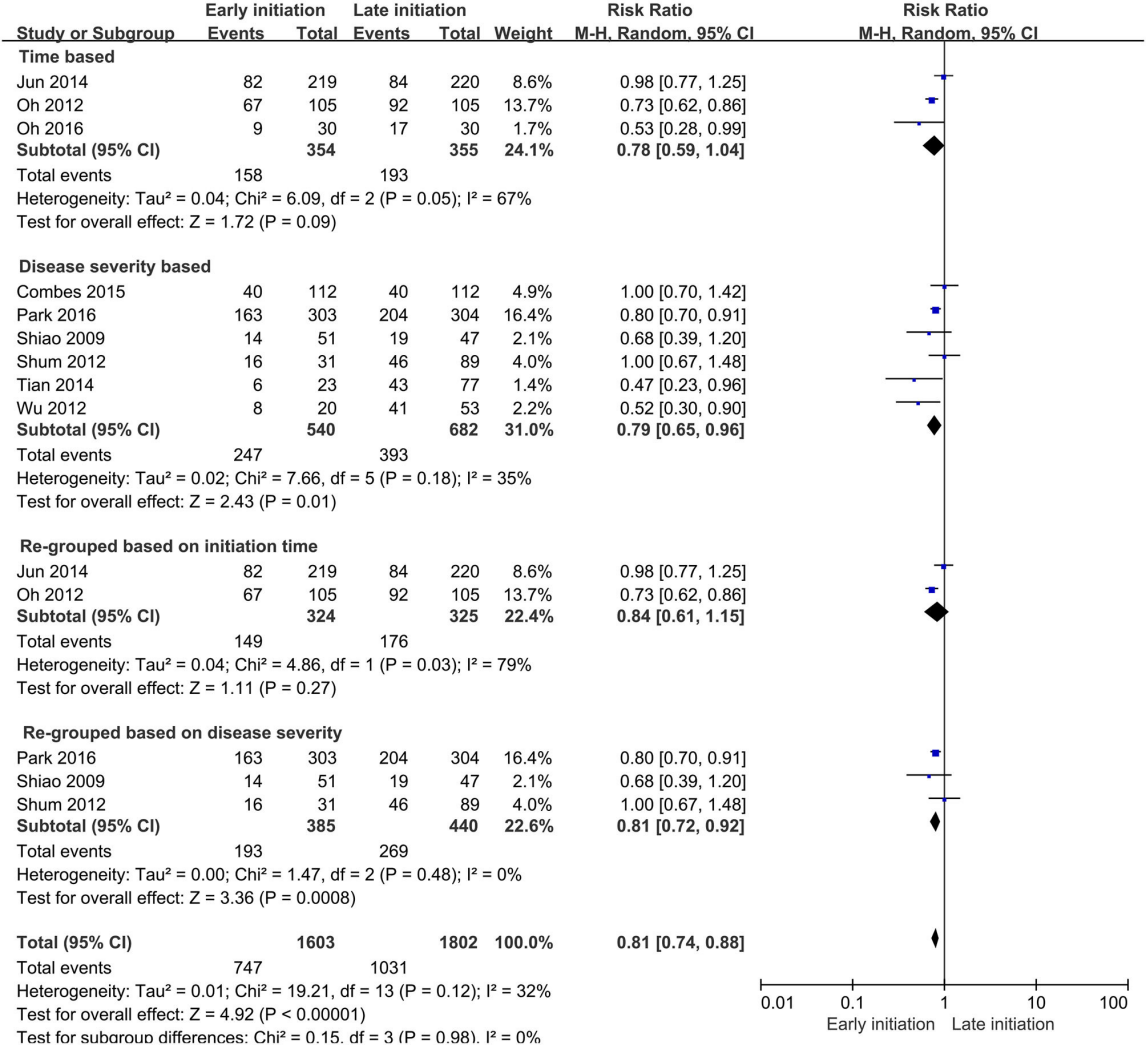
Forest plot for subgroup analysis on 28- or 30-day mortality.

In the subgroup analysis of 28- or 30-day mortality, a similar significant benefit was found in the early initiation of CRRT in the disease severity-based study subgroup (RR 0.79, 95% CI 0.65–0.96, *p* = 0.01) and the re-grouped analysis based on the disease severity subgroup (RR 0.81, 95% CI 0.72–0.92, *p* < 0.001). However, no difference was still found in the time-based study subgroup or the time-based re-grouped subgroup. The heterogeneity analysis showed the same negative outcome in this subgroup analysis (*I*^2^ = 0%, *p* = 0.9, Figure 8)

### Sensitivity and Influence Analysis

The influence of each study was examined by removing one study at a time from the analysis. Any study excluded from the analysis should not change the conclusion or reach a 20% change of the result in the analysis of main outcomes.

## Discussion

This study focused on the initiation timing of CRRT, which is considered as a primary management of patients with AKI in the ICU. The results of 18 studies with 3,914 patients were combined in our meta-analysis, and we found that the early initiation of CRRT could improve the status of the patients with AKI in terms of in-hospital and 28- or 30-day mortality and could be beneficial for renal recovery after CRRT. Furthermore, the subgroup analysis shows a significant benefit of earlier stage of AKI CRRT initiation. To our best knowledge, our study was a very first one to perform this subgroup analysis and put forward the viewpoint that disease severity (AKI stages) should be considered first when evaluating the timing of CRRT initiation.

Timing of CRRT initiation has been discussed over the decades. The actual initiation timing of CRRT in clinical work varies a lot, as it is greatly affected by the subjective judgment of physicians and the distribution of medical resources (36). However, there is still no consensus on whether earlier CRRT initiation can benefit the patients with AKI.

Recently, several large RCTs focused on the CRRT initiation timing (21, 37, 38), which had intensified the discussion of this topic. Our result was consistent with the work of Zarkbock et al. (21), who included 231 patients. They defined early strategy as an initiation of CRRT within 8 h from the diagnosis of AKIN stage 2 and late strategy as initiation within 12 h from diagnosis of AKIN stage 3 and found that early initiation had beneficial effects in 28-, 60-, and 90-day mortalities and RRT dependence after therapy. However, Gaudry et al. (37) and Barbar et al. (38) presented opposite views. Gaudry et al. (37) enrolled 620 patients and randomized them into early and late initiations. The early strategy was indicated as an RRT implementation at the time of randomization and the late strategy was taken under absolute dialysis indicators (in the patients without the indicators shown during ICU stay, RRT was not initiated). The 60-day mortality was measured as the primary outcome in this study, and the authors found no difference in these strategies, but the lowest and highest mortality rates were found in the non-RRT group and RRT group with late initiation, respectively, which coincided with our conclusion. Meanwhile, Barbar et al. (38) enrolled 488 patients and randomized them into early and late strategies with a cutoff time period after randomization for 12 h. The patients were followed for 90 days after RRT, and mortality was measured as a primary outcome; similarly, no benefit was found in the early group. Unfortunately, none of these two studies had given prerequisite data describing the CRRT situations; thus, we cannot take these studies into our meta-analysis, as they might influence our conclusion. We believed that these disagreements mainly come from the definition of early and late that the authors adopted, as in essence was based on the time period. This can also support our view that the timing definition based on the disease severity should be accounted first when discussing CRRT initiation. Other reasons for this disagreement might be the dialysis method chosen in the study of Barbar et al. (38) and no RRT method nor composition of different RRT was described; CRRT as a more positive dialysis method can remove the metabolic wastes continuously and gradually correct the fluid overload. In fact, when the disease was in an early stage, regular hemodialysis rather than CRRT is more likely employed, and this difference in RRT method composition in early and late groups can be the source of the divergence between our study and those of Barbar et al. (38) and Gaudry et al. (39). Theoretically, with appropriate intervention time, CRRT will be more appropriate in maintaining the hemodynamic stability and homeostasis in the patients with AKI and significantly improve the prognosis of the patient and reduce mortality.

Similarly, Gaudry et al. (39) had conducted an meta-analysis and found no benefits of early and late initiation of RRT on 28-day (RR 1.01, 95% CI 0.91–1.13) or 90-day (RR 0.38, 95% CI 0.83–1.16) mortality in 2020, we think this disagreement might also come out from the definition of early and late in the studies they enroll. Most of the early strategy is defined as an RRT conduct at any or late stage of Kidney International Improving Global Outcomes (KIDIGO), RIFLE, and AKIN without serious complications, which is partly contained in our late group. In fact, we believe their negative conclusion is not a symbol saying the timing of RRT initiation is not really important, otherwise, it should remind us to pay further attention to the studies of AKI severity degree of CRRT initiation timing.

This study found benefits of earlier CRRT, especially when its timing was defined by the disease severity, in other words, CRRT was initiated based on an earlier disease stage. In this study, CCRT initiated in patients with AKIN stage 1 or RIFLE-Risk or less stage might lead to a better prognosis for the patients with AKI admitted in ICU. The possible mechanisms might benefit from the lower overall cumulative fluid balance, gentler osmolar shifts, and effective and timely clearance of inflammatory factors and other body wastes, which can prevent the hemodynamic imbalance and further kidney damage in theory (40). Our discussion cannot provide a specific answer, as the mechanisms and pathways still remained to be elucidated.

The heterogeneities across the studies were found in our analyses. Considering that the heterogeneity can arise from different definitions of early and late timing, we conducted the subgroup analysis to assess the effects in the subgroups of time-based studies, disease severity-based studies, studies re-grouped based on time, and studies re-grouped based on the disease severity to measure the effect of early vs. late initiation of CRRT on in-hospital and 28- or 30-day mortalities. However, 60-day mortality, 90-day mortality, and renal recovery after dialysis were beyond this subgroup analysis because not enough trials had provided this information. After the subgroup analysis, the heterogeneity between each trial was known (<75%). Similar results were found in the disease severity-based studies and the studies re-grouped based on the disease severity, while in the subgroups of studies based on time of CRRT initiation and the studies re-grouped based on time, the benefit of early CRRT was not so significant. This may be because the patients requiring early CRRT, as defined by time in observational studies, usually had greater risk and more severe disease and the number of relevant studies was limited.

This study has several limitations. Firstly, only five RCTs were included, the rest were all non-RCTs, and the quality of these studies was limited. Secondly, the publication bias existed because only a small number of studies met our inclusion criteria, which may lead to an overstated beneficial effect of early CRRT on mortality and renal recovery after therapy compared with late CRRT. Thirdly, no meta-regression analysis was conducted to assess the heterogeneity factors. Finally, only mortalities and renal recovery conditions were measured in this study; secondary outcomes, such as length of hospital or ICU stay, vasopressor requirement, bleeding events, and mechanical ventilation were not measured because of the lack of available data.

Although several previous systematic reviews and meta-analyses focused on the RRT timing of the patients with AKI (11, 12, 40, 41), none of these studies focused on CRRT, nor did the trials investigated early and late CRRT separately. This can be a source of heterogeneity. As each of these factors was evaluated in our study, we found that the definition of the initiation timing based on the disease severity may be more effective and beneficial.

Further prospective interventional, large, multicenter trails are necessary to discover the degree of disease severity on the timing of CRRT initiation and to provide more powerful evidence. Further studies should focus on the long-term prognosis to find the relationship between the CRRT initiation timing and chronic kidney disease after AKI.

## Conclusion

Compared with late CRRT, early CRRT can provide a favorable influence on the short-term mortality (e.g., in-hospital mortality and 28- or 30-day mortality) and renal recovery after CRRT, but the effects of different timing of CRRT initiation on long-term mortality remain unclear. Defining initiation timing by disease severity might be more effective. CRRT initiation on the patients with AKIN stage 1 or RIFLE-Risk or less may lead to a better prognosis. We suggest an early stage of AKI disease-CRRT initiation as a beneficial timing choice. Further prospective interventional, large, multicenter trails are warranted to establish the most appropriate initiation timing based on the disease severity and to determine the effectiveness of initiation timing related to the long-term prognosis.

## Data Availability Statement

The original contributions presented in the study are included in the article/supplementary material, further inquiries can be directed to the corresponding author/s.

## Author Contributions

Z-JX, L-yH, and YL conceived the study and wrote the initial protocol and the manuscript. Z-JX and S-YP did the literature search. Z-JX, Q-PZ, and R-JC did the statistical analysis. All authors shared trial data, gave crucial feedback on the protocol, and provided critical revision for and approved the final version of the manuscript.

## Funding

The present work was supported by the National Key Research and Development Program of China (Project No. 2016YFC0906201), the Key Research and Development Program of Sichuan Province (Project No. 2021YFS0166), and the 1.3.5 Project for Disciplines of Excellence, West China Hospital, Sichuan University (Project No. ZYGD18015).

## Conflict of Interest

The authors declare that the research was conducted in the absence of any commercial or financial relationships that could be construed as a potential conflict of interest.

## Publisher's Note

All claims expressed in this article are solely those of the authors and do not necessarily represent those of their affiliated organizations, or those of the publisher, the editors and the reviewers. Any product that may be evaluated in this article, or claim that may be made by its manufacturer, is not guaranteed or endorsed by the publisher.
